# Novel metrics for evaluating the functional coherence of protein groups via protein semantic network

**DOI:** 10.1186/gb-2007-8-7-r153

**Published:** 2007-07-31

**Authors:** Bin Zheng, Xinghua Lu

**Affiliations:** 1 Department of Biostatistics, Bioinformatics and Epidemiology, 135 Cannon Street, Charleston, South Carolina 29425, USA; 2 Laboratory for Functional Neurogenomics, Center for Neurologic Diseases, Harvard Medical School and Brigham and Women's Hospital, Landsdowne Street, Cambridge, Massachusetts 02139, USA

## Abstract

We present the metrics for assessing overall functional coherence of a group of proteins based on associated biomedical literature. A probabilistic topic model is applied to extract biologic concepts from a corpus of protein-related biomedical literature. Bipartite protein semantic networks are constructed, so that the functional coherence of a protein group can be evaluated with metrics that measure the closeness and strength of connectivity of the proteins in the network.

## Abstract 2

Metrics are presented for assessing overall functional coherence of a group of proteins based on the associated biomedical literature.

## Background

A cellular function is usually carried out by a group of proteins, such as the proteins that participate in a common metabolic pathway or a signal transduction pathway. Based on the assumption that the expression of the proteins involved in a biologic process should be coordinated, many computational methods have been developed to identify the potential modules of genes or proteins based on high throughput technologies, such as microarray studies [1-3]. When a candidate protein group is identified algorithmically, it is imperative to evaluate whether the proteins in the group are functionally related, termed the functional coherence of the proteins. Currently, determining the functional coherence of protein groups requires either manually inspection of the associated biomedical literature or utilization of currently available protein annotations. Manually studying of the literature is a labor intensive task and does not scale well with high throughput methodology.

Recently, analyses of gene function annotation, especially in the form of Gene Ontology (GO) [4], have become the most commonly used methods with which to study the function of a list of proteins, and many tools have been developed to perform such analyses (see the recent reviews by Khatri P, Draghici [5] and Curtis and coworkers [6], and the references therein, for details). The GO consists of a set of controlled vocabulary, referred to as GO terms, which has been widely used to describe/annotate proteins in terms of three aspects: molecular function, biologic process, and cellular component. The underlying assumption for GO annotation analysis is that if a group of proteins share similar function or participate in a common cellular process, then they are likely to share GO annotations, such that the terms may be evaluated as 'statistically enriched' within the group. Therefore, the overall function of proteins can be represented by the enriched GO terms.

Although very useful, such analysis has certain drawbacks. First, inconsistency in annotation reduces sensitivity. It is not uncommon for proteins participating in a common metabolic or signal transduction pathway to be annotated with different GO terms because of differing assessments of information by annotators. Such inconsistency makes it more difficult to identify enriched GO terms, thus leading to reduced sensitivity. Second, the approach ignores the relationships among the biologic concepts represented by the enriched GO terms. For example, one may observe enrichment of GO terms GO:0004340 (glucokinase activity) and GO:0004618 (phosphoglycerate kinase activity) simultaneously within a group of proteins. The co-enrichment of these two concepts is biologically meaningful because proteins with these functions participate in a common pathway. However, most of current methods treat enrichment of GO terms as independent events and ignore the biologic importance of the correlation of biologic concepts. Third, when multiple GO terms are 'enriched' within a protein group, it is difficult to derive a quantitative metric to reflect overall functional relationships of the proteins or their statistical significance evaluations. Finally, many statistical methods commonly used to determine the 'enrichment' of GO annotation (for instance, hypergeometric distribution) are sensitive to the size of genome and the frequency of annotations [5,6].

To overcome some of the above-mentioned difficulties, some researchers utilize information on the semantic similarities of GO terms or GO graph structure [7-11] to evaluate the function of protein groups. In these approaches, semantic similarity or GO graph structure are taken into account to evaluate the relationship of GO annotations within a group of proteins. These methods require the proteins of interest to be annotated with GO terms. Currently, however, manual annotation of proteins cannot keep up with the rate of accumulation of biomedical knowledge. Furthermore, there are many organisms whose genomes are not annotated with GO terms, but a body of biomedical knowledge exists in the form of free text.

Instead of relying on GO or other forms of annotations, some researchers directly tap into knowledge in the biomedical literature associated with the proteins, and study their functional relationships through semantic analysis of the literatures. Homanyouni and coworkers [12] and Khatri and colleagues [13] explored the techniques of clustering proteins based on the semantic contents of the biomedical literature associated with the proteins, but the semantic information was not used to evaluate the functional coherence of the proteins *per se*. By mining the biomedical literature associated with proteins, Raychaudhuri and Altman [14] developed a sophisticated scheme and a metric, referred to as neighbor divergence per gene (NDPG), to evaluate the functional coherence of a group of proteins. However, their method requires heuristic setting of multiple parameters and thresholds, whose optimal values may be difficult to determine. Furthermore, their metric is essentially the Kullback-Leibler divergence of two distributions whose value is not normalized; thus, it is difficult to determine the statistical significance of a given score.

In this research, we developed a novel approach to determining the overall functional coherence of a group of proteins. The idea underpinning our approach is that biomedical literature describing a group of proteins that have similar functions or participate in common pathways should share common biologic concepts. This allows us to extract biologic concepts from the literature and to connect proteins through their shared biologic concepts in a bipartite graph, referred to as a protein semantic network (ProtSemNet). In such a graph, the proteins participating in a related function tend to be closely located on the graph. We have designed metrics to measure the functional coherence of a group of proteins by determining their 'closeness' or 'strength of connectivity' on the graph. Furthermore, we have also developed methods with which to evaluate the statistical significance of the functional coherence metrics.

## Results

### Evaluating functional coherence with GO annotation analysis

We first attempted to design metrics based on GO annotation analysis in order to assess the overall functional coherence of protein clusters. (Here we use the terms 'protein cluster' and 'protein group' interchangeably.) The results from this experiment can be treated as a baseline that demonstrates the difficulties associated with this method and provides the motivation for our approach. In this experiment, we collected a set of functionally coherent protein groups and a set of random clusters to evaluate the ability of the GO derived metrics to differentiate the functionally coherent protein groups from the noncoherent ones. For the coherent groups, we selected the protein groups of ten yeast (*Saccharomyces Cerevisiae*) pathways from the Kyoto Encyclopedia of Genes and Genomes (KEGG) database [15]. KEGG is a comprehensive knowledge base that contains information regarding genes and genomes, including a pathway database that describes the known members of cellular pathways. For the noncoherent clusters, we have randomly sampled genes/proteins from the yeast genome and grouped them into clusters with sizes similar to those of the KEGG groups. We employed the most commonly used hypergeometric distribution to evaluate the enrichment of a GO term within a cluster (see Materials and methods, below). We defined a *P *value of 0.05 or less to be statistically significant.

Multiple proteins within a cluster naturally lead to multiple GO terms being associated with the cluster. Contemporary methods evaluate enrichment of each GO annotation independently; this potentially leads to multiple significantly enriched annotations within a cluster. In order to obtain a unified scalar metric for evaluating the functional coherence of the protein group, two intuitive candidate metrics were considered: the number of 'enriched' GO annotations per cluster, and the averaged *P *values of the enriched GO annotations within a cluster. Intuitively, one would expect the first metric to be larger for the functionally coherent proteins, because the proteins in such a cluster are more likely to share GO terms, and the shared GO terms are more likely to be evaluated as 'enriched' than are the nonshared ones. The second metric also makes intuitive sense because if a GO term is enriched as a result of the functional similarity of the proteins, then the *P *values should be more significant than those enriched by random chance.

#### Counting enriched GO terms as a metric

**Figure 1:**
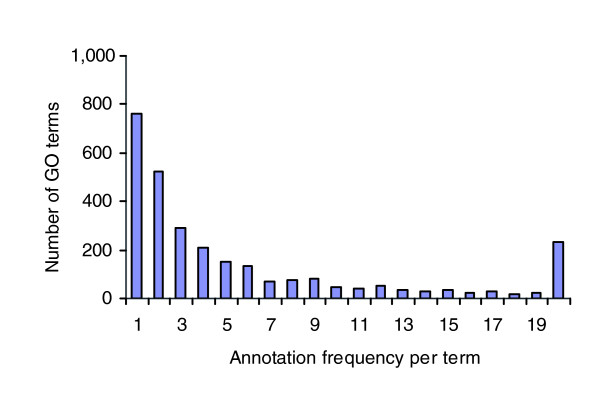
The histogram of GO annotation frequency. GO, Gene Ontology.

When evaluating the 'enrichment' of GO annotation using a hypergeometric distribution, a commonly encountered difficulty is that many low frequency GO terms (for instance, the terms used to annotate only one or two proteins) will be evaluated as 'significantly enriched' whenever they are observed in a cluster with a reasonable size, regardless of whether the cluster is a biologically coherent or a fully random one. To illustrate how often such problem may occur in the real world, we have plotted a histogram of GO term annotation frequency in a recent GO annotation dataset from the yeast genome database (dated 31 March 2007). From Figure 1, one can see that more than 50% of the GO terms appear in the annotation data three times or less. In fact, a large number of GO terms are observed only once in the data. When evaluated with hypergeometic distribution and other methods, these terms exhibit a marked tendency to be evaluated as 'significantly enriched' once they are observed in a cluster. Indeed, all 2,925 but 233 unique GO terms observed in the dataset will be evaluated with *P *< 0.01 if they appear more than once in a cluster of 50 proteins.

As a potential metric for evaluating overall coherence of proteins in a cluster, the number of 'statistically enriched' GO terms in the ten KEGG clusters are collected and compared with those from the randomly drawn clusters. Table 1 shows the averaged number of enriched GO terms per cluster for the two groups. Interestingly, the average number of enriched GO terms in the randomly drawn clusters is higher than that of biologically coherent KEGG clusters. This observation counters the intuition that the more the enriched GO terms exist in a cluster, the more biologically coherent the cluster is. The possible explanation for such a phenomenon is that the functionally coherent protein groups tend to share GO terms, and therefore fewer GO terms are observed. On the other hand, the random groups may tend to contain various GO terms, and some of them are inevitably enriched (as discussed above). Although one can potentially utilize such difference to distinguish a random cluster from a coherent one, by declaring the cluster with fewer enriched annotation as more coherent one, such an approach seems less intuitive and lacks a suitable threshold for making good decisions. For example, is a cluster with zero enriched GO terms more coherent than a cluster with five?

**Table 1:** GO annotation based functional coherence metrics

Group	Average number of 'enriched' GO terms	Average *P *values of the 'enriched' GO terms
KEGG	78.6	0.00078
Random	84.7	0.0023

GO, Gene Ontology; KEGG, Kyoto Encyclopedia of Genes and Genomes.

#### Averaged *P *value as a metric

Another potential metric derived from GO annotation analysis is to determine the average *P *values of the enriched GO terms per cluster, based on the assumption that the *P *values for the enriched GO terms in the coherent clusters may be more significant than those enriched by random chance. Our results indicate that this appears to be the case. Table 1 shows that the average *P *value of the KEGG clusters is indeed smaller (more significant) than that of the random clusters. However, this evaluation also has several drawbacks, as discussed below.

The *P *value for enrichment of a GO term is dependent both on the number of times that the GO term is observed at the whole genome level and on the size of the cluster. For the GO terms with low annotation frequency (for example, GO terms only observed once or twice in the genome), their enrichment tends to be the same in both functionally coherent and random clusters of similar size. Thus, the *P *values of these GO terms do not help in assessing the functional coherence of a cluster, because the 'randomly enriched' GO terms are usually the low frequency GO terms, and they cannot be further enriched in the functionally coherent group. For example, in the glycolysis/gluconeogenesis pathway of yeast (KEGG pathway sce00010), there are several GO terms that are observed only once in the yeast genome annotation (for example, GO:0004332 [fructose-bisphosphate aldolase activity], GO:0004340 [glucokinase activity], and GO:0004618 [phosphoglycerate kinase activity]). The low annotation frequency for these terms is due to the biologic reality that yeast has only one protein performing each of the described functions. However, if these annotations are observed in any randomly grouped cluster of the same size, they will be evaluated as being as 'significant' as in the coherent clusters, because they cannot be further enriched. In addition, because of their rareness, the low frequency GO terms tend to be evaluated with more significant *P *values.

It can be seen that when the average *P *values of the clusters is used to identify the coherent clusters, the results will be determined by the GO terms that have high annotation frequencies at whole genome level and are observed many times within the cluster. In order to find the 'truly enriched' GO term within a cluster, one may have to look for such GO terms manually. During manual searching, one must deal with other difficulties. For example, what should the cut-off annotation frequency be, and what should the cut-off *P *values be? The decision is further complicated by the fact that the enrichment *P *values also depend on the cluster size, and so a comparison of the average *P *values from two clusters with different sizes would be invalid.

Evaluating *P *values of individual GO terms ignores the relationship between the enriched GO terms, which may be more informative than the *P *values *per se*. In the above example of glycolysis/gluconeogenesis pathway, an experience biochemist would discern the relationship among the functions described by those lower frequency GO terms because the proteins with these functions are involved in a biologic pathway. That biochemist would thus reason that the co-occurrence of these terms within a single cluster conveys more information than the individual *P *values, which essentially carry no information in this case. Thus, it is more important to identify the higher level abstraction of protein functions rather than simply counting the GO terms or averaging *P *values. Preferably, one would like to see that a GO term that summarizes the abstract concept of a group of proteins is enriched in the cluster. Indeed, the glycolysis/gluconeogenesis pathway cluster does contain a GO term, namely GO:0006096 (glycolysis). This term is associated with 14 proteins in the genome, and all of them are observed in this KEGG cluster, which should be considered as significantly enriched in the cluster.

It is desirable that all genes are consistently annotated with such a common summarizing GO term, allowing simple evaluation of enrichment and a concept summary. However, the principle adopted by the GO Consortium is to annotate proteins with GO terms as specific as possible, based on available knowledge [4]. Thus, most functionally coherent clusters may not have such a summarizing GO term, but contain a collection of specific terms. To address such difficulty, one may search, manually or automatically, for a GO term that summarizes the information conveyed by the observed specific GO terms. Alternatively, one can directly identify the abstract biologic concept from the literature associated with proteins and use such information to evaluate their functional coherence, without searching for such a 'right' summary GO term. The latter approach will enable us to avoid the annotation bottleneck and the sparse, inconsistent annotation problems.

### Associating proteins with biological concepts

In a previous study we reported the results of identifying/extracting biologic concepts from a protein related corpus from the GO annotation (GOA) [16], using the latent Dirichlet allocation (LDA) model [17]. The results demonstrated that the LDA model was capable of extracting biologically meaningful concepts from the GOA corpus. In essence, a 'topic' identified by the LDA model is a word usage pattern that captures the co-occurrence of words during discussion of concepts and often reflects the abstract concepts conveyed by these words. We applied Bayesian model selection to determine how many topics were suitable to represent the corpus, by choosing the model that fits the corpus with the highest posterior probability *P*(*M*|*D*), where *M *denotes a model and *D *the observed data. A model with 300 topics was found to fit the data well. After inspecting the words associated with the topics extracted using the LDA model, we further removed some topics that did not convey specific biologic concepts but were instead generic (see our supplementary website for the list [18]). A total of 229 topics were retained to construct the ProtSemNet [18].

**Figure 3:**
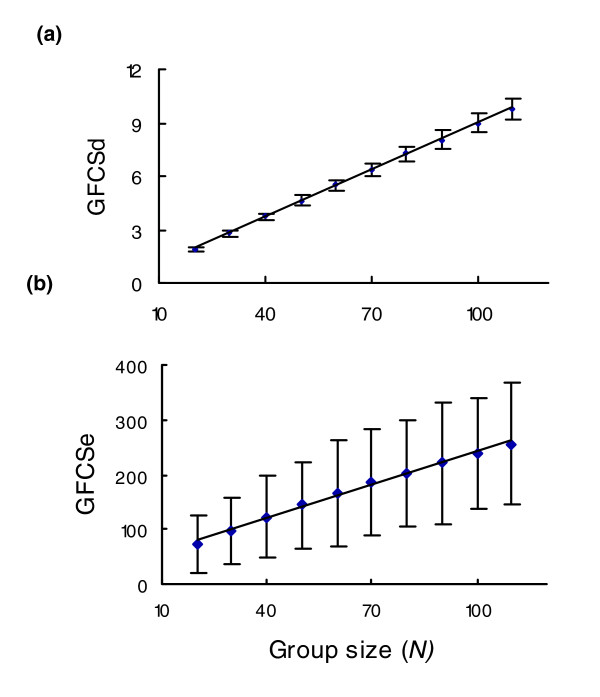
Relationship between groups size and GFCS. GFCS, group functional coherence score.

The LDA model can be used to infer the topic to which each word in a document belongs. Thus, the semantic content of a document can be represented as the presence of topics in that document, and the strength of the topics can be estimated through counting the words belonging to a given topic. See Figure 3 of our previous report [17] for an example of a MEDLINE abstract in which the latent topics for each word is inferred by a LDA model. With such information available, we were able to connect proteins with the semantic topics based on the MEDLINE documents associated with them. Furthermore, the strength of association between a protein and a topic can be represented as the number of words assigned to the topic among all of the documents associated with the protein. Combining the associations between the proteins and the semantic topics, we constructed a protein-topic association matrix, **A**, which can be treated as an adjacency matrix of a weighted, undirected bipartite graph consisting of proteins and topics. We refer to such a graph as a protein semantic network (ProtSemNet). On this network, proteins are connected to each other only through the share biologic concepts, and therefore the proteins sharing similar functions tend to be closely located or strongly connected on the graph.

### ProtSemNets and their properties

We have constructed multiple ProtSemNets consisting of proteins from three well studied species - human, mouse, and yeast - using the proteins from these species. (See Materials and methods, below, for detailed description of the procedures.) The numbers of human, mouse, and yeast proteins contained in the GOA corpus are 7,906, 14,737, and 4,619, respectively. In addition to these species specific ProtSemNets, all proteins in the GOA corpus were mapped to the following unique sets: Cluster of Orthologous Groups (COG) and Eukaryotic Orthologous Group (KOG) [19]. Then, the MEDLINE documents associated with the member proteins of an orthologous group were pooled together, and a unified ProtSemNet consisting of orthologous clusters and biologic topics was constructed, which was referred to as the orthologous ProtSemNet. In order to remove potential noise and reduce computational cost, the element *a*_*pt *_of matrix **A**, whose value was less than 5% of the total number of words associated with a given protein p (the sum of *p*^th ^row of **A**), was set to 0, which is equivalent to removing the edge between protein *p *and topic *t*. When constructing the ProtSemNet, we specified the semantic distance of an edge to be the inverse of *a*_*pt*_, such that the stronger the association between topic and protein, the shorter the distance of the edge. As expected, when connected with thousands of proteins, the 229 biologic topics in the ProtSemNet look like hubs with multiple proteins associated. On the orthologous ProtSemNet, the average degree of connectivity for the biologic topic vertices is 219, whereas the average degree of connectivity for protein vertices is 5.

### Metrics for evaluating functional coherence of a group of proteins

The assumption underlying our approach of evaluating the functional coherence of a group of proteins is that the biomedical literature describing proteins with similar functions should share similar biologic topics, and therefore these proteins should be closely connected on the ProtSemNet. Therefore, the 'closeness' of the proteins on the ProtSemNet graph can be used as a metric for evaluating the functional coherence of the group. Given a ProtSemNet, one can extract a subgraph connecting any arbitrary group of proteins, provided that they are represented in the graph, such that the total semantic distance of the subgraph is shortest. A subgraph satisfying such a requirement is a tree, and the problem of identifying such a tree is referred to as the Steiner tree problem [20]. With a Steiner tree for a group of available proteins, we designed two metrics as the group functional coherence score (GFCS): the total number of edges of the Steiner tree, referred to as GFCSe; and the total semantic distance of the Steiner tree, referred to as GFCSd. The interpretation of the values is as follows; a small value for GFCSe (or GFCSd) indicates close (or strong) connections among the proteins in the group.

Based on the assumption that a functionally related group of proteins should be located closely on a ProtSemNet, one would expect that the scores for such a group of proteins would be significantly different from those of the protein groups consisting of randomly picked proteins from the same ProtSemNet. Thus, statistical methods can be developed to compare the significance of the scores of a group of interest with the scores of randomly picked protein groups. More specifically, we should like to evaluate whether the GFCS scores from a cluster of interest are statistically significantly smaller than those from the randomly picked protein groups. To this end, one can think of the random GFCS scores as being determined by a distribution, and statistical inference approaches can be applied to estimate the parameters for the distribution. Once the distribution for the random score of a given ProtSemNet and a given cluster size is defined and the estimated parameters are available, one can access the statistical significance of the GFCS score from any arbitrary protein group from the ProtSemNet with respect to the random score distribution. Estimation of parameters can be achieved through a simulation process in which a large number of random protein groups can be generated and used as the samples for estimating the distribution parameters. Note that a GFCS score distribution is not only specific for a given ProtSemNet but it is also specific to a given cluster size, and therefore the estimation process should accommodate different distributions and take the cluster size into account.

**Figure 2:**
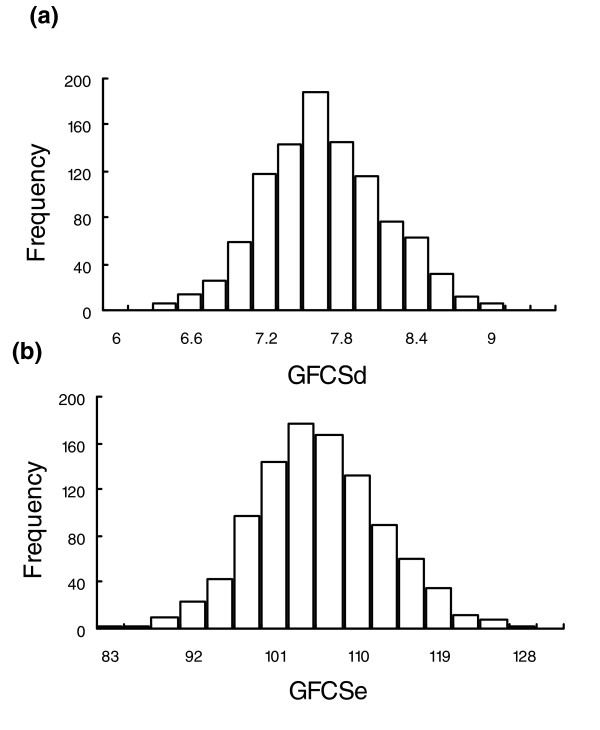
Distributions of GFCS scores. Showsn are plots of the the histograms of **(a) **GFCSd and **(b) **GFCSe scores from 1,000 random clusters, each containing 50 proteins, drawn from the mouse ProSemNet. GFCS, group functional coherence score.

To estimate the parameters for the random GFCS score distributions, we have randomly drawn protein groups of various sizes from a ProtSemNet of interest. For each cluster size, say 50 proteins, we collect 1,000 random protein groups. Therefore, the scores from these groups can be treated as samples from the random score distribution, and the parameters for such a distribution can be estimated based on these samples (see Materials and methods, below, for details). Figure 2 shows the distribution of the GFCSe and GFCSd for 1,000 protein groups consisting of 50 orthologous proteins randomly picked from the orthologous ProtSemNet. The distributions for the scores closely follow the shape of the normal distribution. This phenomenon is due to the fact that the GFCSs are the sums of the weights of many edges and, according to the central limit theorem [21], such a variable will be assume a normal distribution if the number of edges is sufficiently large. Thus, the probability of observing a given score or less, the *P *value, can be determined according to a normal distribution with estimated mean and variance.

To correct for the dependence of GFCSs on cluster size, a linear regression model is estimated for each of the four ProtSemNets to capture the relationship between each of GFCSs and the group size N. Figure 3 shows the linear relationship between group size and the GFCSe and GFCSd for random groups from the orthologous ProtSemNet, with regression coefficients (R^2^) of 0.9998 and 0.9955, respectively. The results indicate a good linear relationship exists between cluster size *N *and GFCSs, and all four ProtSemNets exhibit strong linear relationships with varying estimated parameters.

### GFCSs as metrics evaluating functional coherence

To test whether GFCSs can correctly differentiate the coherent protein groups from randomly picked groups, we selected 30 pathways for human, mouse, and yeast from the KEGG database [15] as the functionally coherent protein clusters and evaluated whether their GFCSs are significantly different from the distributions for the random protein groups. The GFCSs for the KEGG clusters were evaluated using both the species specific ProtSemNets and the orthologous ProtSemNet. Table 2 shows the results for 12 KEGG pathways for which the GFCSs are determined from the species specific ProtSemNets, and additional results for all groups are available at our supplementary website [18]. From Table 2, we can see that if a *P *value of 0.05 is deemed significant, then all KEGG groups have statistically significant GFCSe scores, indicating that the method has correctly detected that the members of these groups are not randomly picked from the network.

**Table 2:** GFCS evaluated from species-specific ProtSemNet

KEGG pathway	GFCSe	*P*	GFCSd	*P*
Apoptosis (hsa04210)	86	9.35 × e^-8^	5.36	1.59 × e^-8^
Glycolysis (hsa00010)	68	1.62 × e^-10^	7.29	0.84^a^
Focal adhesion (hsa04510)	174	1.56 × e^-14^	12.48	7.51 × e^-5^
JAK-STAT (hsa04630)	147	2.52 × e^-27^	12.11	1.56 × e^-05^
ATP synthesis (mmu00190)	33	3.01 × e^-08^	3.42	1.17 × e^-04^
Calcium signaling (mmu04020)	102	0.04	9.24	2.91 × e^-6^
Actin regulation (mmu04810)	122	3.37 × e^-5^	7.47	6.48 × e^-13^
Cytokine receptor (mmu04060)	176	1.65 × e^-39^	15.62	0.99^a^
Purine metabolism (sce00230)	111	3.69 × e^-4^	8.33	0.34^a^
MAPK (sce04010)	78	5.65 × e^-8^	3.15	5.61 × e^-8^
Ribosome (sce03010)	113	1.61 × e^-26^	16.58	0.999^a^
Oxidative phosphorylation (sce00190)	74	1.6 × e^-12^	5.42	0.001

GFCS, group functional coherence score; JAK, Janus kinase; KEGG, Kyoto Encyclopedia of Genes and Genomes; MAPK, mitogen-activated protein kinase; ProtSemNet, protein semantic network; STAT, signal transducer and activator of transcription. ^a^Non-significant *P *value.

**Figure 4:**
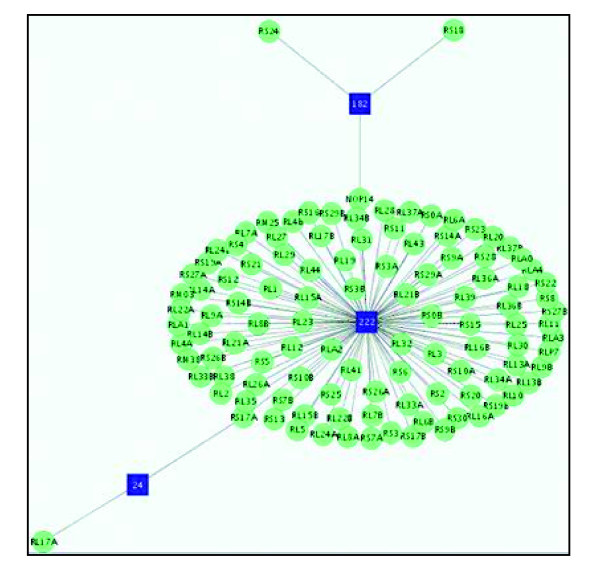
The Steiner tree of the yeast ribosome pathway. A protein is represented a circle while a topic is represented as a box. Topic 222 is related to ribosome.

On the other hand, although most of the GFCSd scores are significant, there are four groups whose scores are not significant. We further investigated the results for one of these pathways, the ribosome pathway (KEGG sce03010). As shown in Figure 4, the LDA correctly identified that the concept 'ribosome' was the major topic for the proteins in this group. Therefore, most proteins formed a cluster around this topic in the Steiner tree. To further investigate why the GFCSd score for this tight group is nonsignificant, we traced all the proteins and their associated MEDLINE records. We noticed that most of proteins in this pathway are associated with only one MEDLINE record; thus, the total number of words associated with the major topics for these proteins tend to be smaller than most protein-topic associations. Recall that the semantic distance, *w*_*pt*_, of a protein *p *to a biological topic *t *is calculated as the inverse of the number of words in the documents associated with the protein and the topic. Thus, if the number of documents associated with a protein is small, then the semantic distance tends to be large. This indicates that the GFCSd is highly sensitive to the number of documents and, in turn, the number of words associated with a given protein in the GOA corpus. We conjecture that one reason for such imbalanced annotation is that the annotators do not cite all of the papers for the proteins with well known function, rather than resulting from a lack of documents describing the proteins. Such bias can be avoided by developing a technique to associate proteins automatically with the relevant literature or to devise a normalized semantic distance metric. We have not fully investigated the other three nonsignificant clusters, but we conjecture that the same reasoning might account for the nonsignificant *P *values.

**Figure 5:**
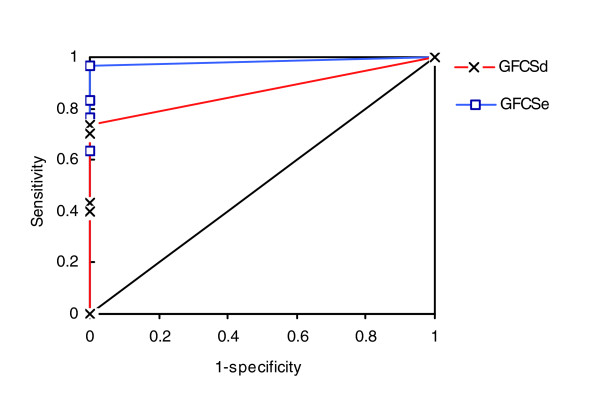
ROC curves for GFCSe and GFCSd. GFCS, group functional coherence score; ROC, receiver operating characteristic.

We further used the sensitivity, specificity, and receiver operating characteristic (ROC) analysis [22] to evaluate the discriminative power of the GCFSs obtained from the species specific ProtSemNet. For this experiment we randomly draw 30 protein groups, with sizes similar to those from KEGG pathways, from the human, mouse, and yeast ProtSemNet, respectively. If the significance threshold *P *value is set at 0.05, the sensitivity and specificity for GFCSe are 0.97 and 1.0, respectively, and the sensitivity and specificity for GFCSd are 0.73 and 1.0, respectively. Using random groups as negative cases and the KEGG pathway groups as positive cases, we progressively set the significance threshold at 1 × e^-4^, 1 × e^-3^, 5 × e^-3^, 1 × e^-2^, and 5 × e^-2 ^to perform ROC analysis. Figure 5 shows the ROC curves for both GFCSe and GFCSd. The results indicate that the metrics have excellent discriminative power, with the area under the ROC curve being 0.98 and 0.86 for GFCSe and GFCSd, respectively.

### Pooling knowledge from multiple species

Our results indicate that the GFCSs, especially the GFCSe, obtained from the species specific ProtSemNet are capable of distinguishing the functionally coherent (nonrandom) protein groups from the randomly produced protein groups. Beyond the species specific ProtSemNet, we believe that it would be advantageous to use the ProtSemNet as a tool to pool knowledge from different species and use the collective information to evaluate protein functional coherence. The key advantage is that it will allow us to evaluate the functional coherence of the proteins from species that are not well studied, through mapping them to orthologous clusters. We constructed an orthologous cluster ProtSemNet and re-evaluated the GFCSs for the protein groups (see Materials and methods, below, for details). Table 3 shows the scores and *P *values evaluated using the orthologous ProtSemNet for the same pathways in Table 2. It is notable that *P *values for many GFCSd become more significant (decrease), indicating that pooling information alleviated the bias caused by sparse annotation and strengthened the relationships among the protein and semantic topics. Although the *P *values for the GFCSe scores do not diminish uniformly, the score retains the discriminative power because all of the *P *values for the KEGG pathways are statistically significant.

**Table 3:** GCFS evaluated from the orthologous ProtSemNet

Protein pathway	GFCSe	*P*	GFCSd	*P*
Apoptosis (hsa04210)	72	2.54 × e^-10^	0.77	3.12 × e^-9^
Glycolysis (hsa00010)	54	0.0062	0.83	4.50 × e^-4^
Focal adhesion (hsa04510)	99	1.66 × e^-7^	0.58	1.34 × e^-14^
JAK-STAT (hsa04630)	153	2.19 × e^-07^	3.11	7.48 × e^-11^
ATP synthesis (mmu00190)	71	2.14 × e^-4^	0.83	4.28 × e^-07^
Calcium signaling (mmu04020)	76	4.43 × e^-8^	0.40	1.16 × e^-12^
Actin regulation (mmu04810)	80	0.0089	0.53	7.45 × e^-9^
Cytokine receptor (mmu04060)	199	2.63 × e^-8^	4.51	4.44 × e^-12^
Purine metabolism (sce00230)	130	2.07 × e^-4^	2.57	6.92 × e^-19^
MAPK (sce04010)	101	0.0069	1.76	1.76 × e^-05^
Ribosome (sce03010)	102	9.50 × e^-25^	7.19	0.471^a^
Oxidative phosphorylation (sce00190)	92	1.41 × e^-6^	2.87	0

GFCS, group functional coherence score; JAK, Janus kinase; MAPK, mitogen-activated protein kinase; ProtSemNet, protein semantic network; STAT, signal transducer and activator of transcription. ^a^Non-significant *P *value.

### Connecting topics with proteins

**Figure 6:**
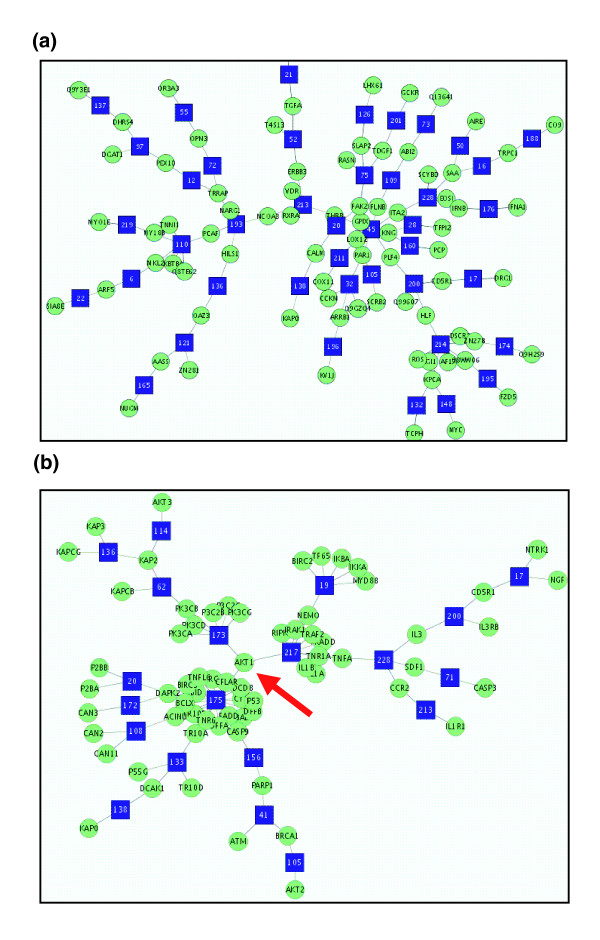
Steiner trees for random and KEGG protein groups. The biologic topics are represented by square vertices, whereas proteins are represented by circle vertices. **(a) **A Steiner tree of a random protein group. **(b) **The Steiner tree of human apoptosis pathway proteins. KEGG, Kyoto Encyclopedia of Genes and Genomes.

Figure 6 shows examples of the Steiner trees for a randomly selected group of 50 proteins (panel a) and for the human apoptosis pathway (panel b) extracted from the human ProtSemNet. Panel b shows that proteins in the human apoptosis pathway tend to form clusters around the topics, especially four topics, namely 175, 173, 217, and 19, which have more than five associated proteins. By checking the high probability words for these topics, they can be summarized as follows: apoptosis for topic 175; phosphoinositide 3-kinase for 173; tumor necrosis factor pathway for 217; and platelet-derived growth factor pathway for 19. Interestingly, protein Akt1 (indicated by an arrow in Figure 6b) connects three major topics in this group, which agrees well with biologic knowledge. Thus, the Steiner tree extracted from the ProtSemNet not only clusters the proteins with similar functions but also brings related biologic topics together. In fact, we found that many proteins within a functionally coherent group are more likely to serve as bridges between topics within the Steiner tree and random groups (data not shown).

## Discussion

In a cell, multiple proteins usually work closely to perform cellular functions, for example proteins in a metabolic pathway. One major research area in bioinformatics focuses on identifying such protein 'modules' based on functional genomic or proteomic data via computational approaches. Once a tentative module is identified, it is imperative to evaluate whether the members of this module really are functionally connected and worthy of further investigation. In this study, we designed and evaluated novel metrics with which to evaluate the functional coherence of a group of proteins. These metrics take into account not only the common shared functions of a group of proteins but also the relationships among these functions via a network analysis approach.

### Connecting proteins through biologic concepts

By extracting the biologic concepts from the literature associated with proteins and constructing ProtSemNets, our method effectively connects proteins through their shared functions. The bipartite network not only groups proteins according to function description, but it also establishes connections between biologic concepts via proteins. Connecting proteins via biologically meaningful semantic topics in the literature has the following advantages. First, it allows us to evaluate the 'functional closeness' of proteins without requiring them to interact physically, which is sensible in that proteins involved in a pathway do not necessarily bind to each other physically. Second, it does not require proteins to be co-mentioned within the same biomedical article in order to establish connections. Thus, it overcomes a difficulty encountered by other natural language processing or information extraction approaches [23] that require proteins to be co-mentioned in order to establish associations. Third, the multiple topic nature of the LDA model captures the multifaceted character of proteins; for example, a protein can be part of an electron-carrier chain in mitochondria and be involved in the cellular process of apoptosis. Thus, such proteins provide connections between biological concepts. Finally, our method does not require manual annotation like GO does, which can be a bottleneck to accumulation of knowledge. Also, it overcomes the limitation of GO that concepts from one domain of GO (for example, molecular function) cannot be connected to concepts of other domains (such as cellular component).

The ProtSemNet fulfills both goals of connecting functionally related proteins through shared functions and bridging the biologic functions through proteins. As demonstrated in the example of the human apoptosis pathway (Figure 6b), the proteins are closely connected by their functional descriptions, such as apoptosis, phosphoinositide 3-kinase, chromatin structure, and tumor necrosis factor pathway. Furthermore, a Steiner tree consisting of functionally coherent proteins brings several biologically related biological concepts together, for example that activation of the tumor necrosis factor pathway will activate apoptosis, which involves destruction of chromatin structure and DNA fragmentation. Therefore, this approach not only provides a means with which to evaluate the functional coherence of proteins but it also explains the connections among the proteins associated with a seemingly wide range of biologic concepts. This approach overcomes the shortcomings of current methods that treat the enrichment of protein functions within a group as independent [5,6]. Constructing the ProtSemNet with the orthologous clusters and biologic concepts builds a foundation for knowledge enhancement, because such a network effectively pools the knowledge regarding orthologous groups from different organisms. This network allows one to connect proteins, including those in species that are not well studied, to biologic concepts and in turn to other proteins, thus potentially leading to discovery of functions of previously unknown proteins.

In this study, biologic concepts are automatically extracted using the LDA model, and a Bayesian model selection approach was employed to determine the number of topics in order to avoid overfitting of training data. The extracted topics are well distinguishable, although some of them tend to represent high level concepts. One advantage of extracting biologic concepts in an automatic (unsupervised) manner is the avoidance of expensive manual construction of a protein semantic network, and the automatic approach potentially provides more consistent associations between proteins and biologic concepts. However, because the approach is unsupervised, the quality of the ProtSemNet is limited by the quality and granularity of the semantic topics extracted by the LDA model.

### Determining the functional coherence

Once a ProtSemNet is constructed, either a species specific or an orthologous ProtSemNet, it allows us to evaluate the compactness of the subgraph connecting a group of proteins with unified scores and, more importantly, to determine the statistical significance of the functional coherence scores. Based on the experimental results presented here, we believe that the GFCSe is a more sensitive and robust metric than is GFCSd. The GFCSe can correctly capture strong connections between a protein and its major topics. Furthermore, the quantity of the score is not sensitive to variance in the number of documents associated with a given protein. Such variance can be introduced due to the availability of literature and/or the biases in annotating proteins (some proteins are annotated more extensively than others). The GFCSd appears to fall prey to such variance and fails to identify the group of proteins that are known to be functionally coherent. However, if automatic information retrieval techniques are employed to identify large amounts of biomedical literature associated with proteins, then this problem can potentially be alleviated. In addition, we have also observed that many proteins in the KEGG pathways do not have GO annotations in the GOA data, and so they are not represented in the ProtSemNet. These observations indicate that the current manually annotated databases can not keep up with the rate of accumulation of biomedical knowledge, and there is a need for more extensive and automatic information retrieval methods to systematically associate proteins with biomedical literature for comprehensive representations of biomedical knowledge.

### Semantic analysis with LDA

In this research, we directly relate proteins to the semantic concepts from the biomedical literatures and utilize such relationships to determine the closeness of the semantic information of proteins as metrics for evaluating the functional coherence of any group proteins. Directly utilizing the semantic information from the biomedical literature allows us to avoid the potential difficulties associated with the sparse annotation phenomenon and the annotation bottleneck. Other closely related research utilizing semantic information to evaluate protein functional coherence is the NDPG metric proposed by Raychaudhuri and Altman [14]. However, the lack of available software with which to evaluate NDPG prevents us from directly comparing the two methods.

Semantic analysis using LDA model has the following advantages over the conventional semantic analysis. First, it accommodates the fact that a protein can be associated with multiple biologic processes, and so its associated literatures may consist of multiple topics. This allows proteins that share a common biologic concept to be closely related on the ProtSemNet, without requiring all other biologic aspects of the proteins to agree. Second, the LDA model allows us to represent a protein in a semantic concept space, rather than in the vocabulary space. Such capability allows us to associate proteins as long as their associated literatures share a similar concept, without requiring the similar composition of words in the literatures, thus increasing the sensitivity of detecting connections. Third, our approach provides metrics whose distributions are well behaved, which enables us to estimate the statistical significance of the scores.

## Conclusion

In this research we demonstrate that the metrics based the semantic similarity of the biomedical literature associated with proteins can be used to evaluate the functional coherence of the proteins. We have also demonstrated that the amount of information represented in the training corpus is critical to the usefulness of our method. One future direction of research is to retrieve information beyond the manually annotated training corpus. With advances in natural language processing and information retrieval technologies, it is possible to retrieve protein related literature, identify the protein entities, and extract relevant information at a large scale, and more comprehensive information may provide better evaluations.

## Materials and methods

### Evaluation of enrichment of GO annotations

For this experiment, we used the 31 March 2007 version of GO annotation data for the yeast *Saccharomyces cerevisiae *from the GO consortium website. Let *M *denote the total number of proteins in this dataset, let *K *be the number of times a GO term is observed in the annotation data, let *n *be the size of a cluster, and let *x *be the number times that the GO term is observed in the cluster. Assuming that *x *is distributed as a hypergeometric distribution [21], the probability of observing *x *can be evaluated as follows:



### Dataset

The GOA annotation data (version 28.0) from the GOA project [16] were downloaded from the European Bioinformatics Institute. In this dataset, the proteins from the Uniprot database [24] are annotated with GO terms. Many of these GO annotations are associated with a PubMed identification number (PMID), indicating sources of information for the annotations. This dataset provides a bridge between proteins and their associated literature. We extracted 26,084 PMIDs from the dataset and retrieved the corresponding MEDLINE titles and abstracts through the batch service provided by the National Center for Biotechnology Information (NCBI). MEDLINE references totaling 26,084 were retrieved. There are 39,336 proteins associated with this document set.

The documents were pre-processed by removing 'stop words' (see our supplementary website [18]) and stemming. There is a total of 52,350 unique terms in this corpus. We trimmed this vocabulary by removing terms deemed less relevant to biology. In order to determine whether a word was relevant to biology, we calculated the mutual information (MI) of a word with respect to the GO terms associated with the corpus. The MI is determined as follows:

$$\text{MI}(\text{w},\text{g})=\sum_{\text{w},\text{g}\in \{0,1\}}\text{p}(\text{w},\text{g})\log\frac{\text{p}(\text{w},\text{g})}{\text{p}(\text{w})\text{p}(\text{g})}$$

where *p*(*w*,*g*) is estimated by counting the documents in which a word *w *and a GO term *g *are present or absent (1 or 0, respectively). The biologic relevance of a word is determined as its maximal MI with respect to any GO term. When the words were sorted in descending order according to their MI, we found that words with low MI tended to be either very common or very rare words. The final vocabulary list contained 18,725 unique terms. The corpus is hereafter referred to as the GOA corpus.

### Extracting semantic topics using the LDA model

We applied the LDA [17,25,26] to extract a set of common biologic concepts from the GOA corpus. The LDA is a latent variable model, treating a document as a mixture of words from multiple topics. It simulates the process of 'generating' a text document with following steps. First, choose a *T *dimensional topic content vector θ, which is a parameter vector for a multinomial distribution, from a Dirichlet distribution with parameter α, where *T *is the number of topics. Second, for the *n*^th ^word in the document, choose a topic *z*_*n *_from a multinomial distribution governed by θ. Third, conditioned on its topic *z*_*n*_, choose the word *w*_*n *_from a multinomial distribution governed by a parameter vector ϕ_*zn*_. Finally, the parameters ϕ are distributed as Dirichlet governed by β. Provided with a collection of text documents, the LDA can infer the latent topic variable for each word and extract the word usage patterns ϕ, which closely relate to human-understandable topics. Furthermore, for each word *w *in the corpus, the LDA model infers its latent topic variable *z *using a Gibbs sampling algorithm described in detail elsewhere [17,25]. We further applied a Bayesian model selection approach [17,25] to determine the 'optimal' number of topics that represent the corpus well. Thus, after inference of the topic assignment of each word in a document, the semantic contents of the document can be represented with counts of words for each topic.

### Mapping proteins to orthologous groups

In order to pool the knowledge accumulated from different organisms, we map proteins from different organisms to a unified set of orthologous clusters. An orthologous group of proteins consists of proteins from different species that evolved from a common ancestor. Usually, the orthologous proteins have the same or similar functions. The STRING database [27] maintains information on interacting proteins, in which interactions are defined as either direct physical binding or participation in a common pathway. In addition, it also assigns each protein in the database to the cluster of orthologous group (COG) [19] or the eukaryotic orthologous group (KOG).

To map the proteins associated with the GOA corpus to COG/KOG, the following steps are taken. First, the COG/KOG id was retrieved if the protein of interest was in the STRING database. Second, if the protein of interest was not in the STRING database, then a BLAST search against the STRING database was performed to find the most similar sequences, and these COG/KOG ids was transferred to the protein. The criteria to assign a protein to a COG/KOG were adopted from the STRING database [27], in which the protein sequence should have a significant BLASTP *e*-value (≤10^-6^) with entry proteins in STRING and the first three hit sequences should have the same COG/KOG id. Finally, if none of above conditions was satisfied, then a protein was treated as a sole member of a new orthologous group, and this new group was added to the collection of orthologous groups. As a result, the union of the observed COG/KOG and the newly assigned groups constitute a total of 12,101 orthologous protein groups, and all 36,151 proteins from the GOA corpus were assigned to one of these groups.

### Constructing a protein semantic network

In the GOA corpus, each document is associated with one or more proteins. With the biologic topics in the documents being inferred by the LDA semantic analysis, associations between the biological semantic topics and the proteins can be established. A *P *× *T *matrix protein semantic topic association matrix **A **was constructed such that the element *a*_*pt *_represents the count of words that are assigned to the topic *t *in all the documents associated with the protein *p*. The matrix **A **can be thought of as an adjacency matrix for a bipartite graph. A bipartite graph consists of two types of vertices, such that only the edges connecting vertices of different types are allowed, but not edges joining vertices of the same type. In our case, the two types of vertices are proteins and biologic concepts. We used the matrix **A **to construct a weighted undirected bipartite graph *G *= (*V, E, W*). In this graph, the vertex set *V *consists of the union of proteins and biologic topics, the edge set *E *consists of connections between proteins and topics, and the weight set *W *consists of weights (distance) associated with the edges. We define an edge between a protein *p *and a topic *t *if the element *a*_*pt *_of matrix **A **is nonzero. The weight of the edge *w*_*pt *_is defined as the inverse of *a*_*pt*_, which is referred to as semantic distance between the protein and the topic. That is, the more words associating a protein to a topic, the shorter the distance between the two vertices. We refer to such a bipartite graph as a protein semantic network (ProtSemNet).

### Steiner tree and group functional coherence score

For a weighted undirected graph *G *= (*V, E, W*), consisting of a collection of vertices *V*, and a set of edges *E *and their associated weights *W*, the Steiner tree problem is defined as follows. Given a subset of vertices *V*_*s *_of *G*, return a subgraph *G*_*s *_such that all the vertices in *V*_*s *_are connected by *G*_*s *_and the total length of *G*_*s *_is the minimum among all possible subgraphs connecting *V*_*s*_. By the latter requirement, *G*_*s *_will be a tree with vertices of interest being the leaf nodes. The Steiner tree problem is an NP complete problem, but numerous approximate algorithms are available.

In this study, Kou's algorithm [20] was adopted to extract an approximate Steiner tree. The outline of the algorithm is as follows. First, given a set of proteins, find all pair-wise shortest paths on the ProtSemNet between all possible pairs of the proteins of interest. Construct a complete undirected weight graph *G*_*comp*_, in which a protein is fully connected to the rest of the group. Second, find the minimal spanning tree *T*_*c *_of *G*_*comp*_. Third, construct a graph, *G*_*s*_, by replacing the edges of *T*_*c *_with the corresponding path in the ProtSemNet. Fourth, find the minimal spanning tree, *T*_*s*_, of *G*_*s *_by repeating step 2. Fifth, construct a Steiner tree, *T*_*st*_, from *T*_*s *_by removing unnecessary edges in *T*_*s*_, so that all the leaves of *T*_*st *_are in the set of proteins of interest.

With a Steiner tree for a group of available proteins, the number of edges and the total distance of the tree are used as two metrics for the group functional coherence scores, referred to as GFCSe and GFCSd, respectively, or collectively as GFCSs. Note that the smaller the GFCSs, the more compactly connected is the group of proteins, and the more functionally coherent is the group.

### Evaluating statistical significance of a GFCS

When a ProtSemNet is constructed, the samples of random protein groups were drawn from the proteins on the graph and the Steiner tree for each random group were extracted. For each group size, ranging from 10 to 150, with a step of 5, a collection of 1,000 random groups was sampled. The corresponding Steiner trees and their GFCSs were collected. For a given group size, these 1,000 samples were used to estimate means and variances of the GFCSs. Furthermore, because a GFCS is dependent on the group size *N*, linear regression models between the group size *N *versus the means of GCFSe and GFCSd were estimated. With the estimated regression parameter available, the expected mean GFCS for a random group with a given size *N *can be determined using the linear equation and the variance of the sample group with the size N* closest to *N*.

When a new GFCS* of a group of proteins with group size N* is given, we calculated the probability of the observing GFCS* or less if it was drawn from the population of the random protein groups with the same group size - the *P *values for the GFCS*. According to the central limit theorem, the sum of *N *random independent variables from any arbitrary distribution will be asymptotically distributed as a normal distribution as *N *approaches infinity. Thus, the *P *value of observing GFCS* from a population of random proteins can be approximately determined according to a normal distribution function, provided with a mean and variance of the distribution.

### Supplementary data

Supplementary data are available from our website [18].
